# Importance of activated leukocyte cell adhesion molecule (ALCAM) in prostate cancer progression and metastatic dissemination

**DOI:** 10.18632/oncotarget.27279

**Published:** 2019-10-29

**Authors:** Andrew J. Sanders, Sioned Owen, Liam D. Morgan, Fiona Ruge, Ross J. Collins, Lin Ye, Malcolm D. Mason, Wen G. Jiang

**Affiliations:** ^1^ Cardiff China Medical Research Collaborative (CCMRC), Division of Cancer and Genetics, Cardiff University School of Medicine, Cardiff, UK; ^2^ Division of Cancer and Genetics, Cardiff University School of Medicine, Cardiff, UK; ^3^ Alfred Russel Wallace Building, Upper Glyntaff, University of South Wales, Pontypridd, UK

**Keywords:** ALCAM, prostate cancer, bone, metastasis, serum biomarker

## Abstract

Activated Leukocyte Cell Adhesion Molecule (ALCAM) has been linked to the progression of numerous human cancers, where it appears to play a complex role. The current study aims to further assess the importance of ALCAM in prostate cancer and the prognostic potential of serum ALCAM as a biomarker for prostate cancer progression. Here we demonstrate enhanced levels of tissue ALCAM are associated with metastasis. Additionally, elevated serum ALCAM is indicative of progression and poorer patient outlook, and demonstrates comparable prognostic ability to PSA in terms of metastasis and prostate cancer survival. ALCAM suppression enhanced proliferation and invasiveness in PC-3 cells and motility/migration in PC-3 and LNCaP cells. ALCAM suppressed PC-3 cells were generally less responsive to HGF and displayed reduced MET transcript expression. Furthermore a recombinant human ALCAM-Fc chimera was able to inhibit LNCaP cell attachment to HECV and hFOB1.19 cells. Taken together, ALCAM appears to be a promising biomarker for prostate cancer progression, with enhanced serum expression associated with poorer prognosis. Suppression of ALCAM appears to impact cell function and cellular responsiveness to certain micro environmental factors.

## INTRODUCTION

Dissemination and metastatic spread of cancer cells is a key determinant of patient prognosis and the bone is a common site for metastasis arising from prostate cancer [1]. Additional research is required to fully comprehend the molecular and cellular mechanisms involved in these processes and to develop new therapeutic strategies.

Activated leukocyte cell adhesion molecule (ALCAM, CD166) was initially characterized by Bowen *et al.* in 1995 [2]. ALCAM, a transmembrane glycoprotein, is a member of the immunoglobulin superfamily and has been identified as mediating homophilic, ALCAM-ALCAM, and heterophilic, ALCAM-CD6, interactions [2, 3]. ALCAM has been identified as a substrate of a disintegrin and metalloprotease (ADAM) 17 and can be shed from the cellular surface, a process that can be enhanced by epidermal growth factor (EGF) and transforming growth factor (TGF) β [4–6]. ALCAM has been implicated to influence cellular traits associated with cancer progression *in vitro* and *in vivo* [6–11], though there is some conflict within the literature. Alterations in ALCAM expression have been reported and associated with the progression or prognosis of various human cancers including, breast [7, 12–15], melanoma [16, 17] and gastric [18, 19] cancer, however there are again contrasting reports within the literature.

Accumulating evidence suggests that ALCAM may play a role in cancer cell dissemination and development within the bone environment. Early work has demonstrated reduced ALCAM levels in breast cancer patients who developed skeletal metastasis [14]. Additional studies exploring the prognostic role of ALCAM in breast cancer dissemination have implicated over-expression of ALCAM with nodal involvement and a tendency toward increased tumor cell presence in the bone marrow [7]. Hansen *et al.,* have explored the role of ALCAM in prostate cancer [6]. Using a number of *in vivo* models they demonstrated that ALCAM suppression does not impact on growth or local invasion of cancer cells inoculated into the prostate but significantly reduced skeletal metastasis and burden following intracardiac inoculation and resulted in reduced growth and survival of intratibially inoculated cells [6].

The current study aims to further explore the functional role of ALCAM in regulating aggressive traits in prostate cancer cells and their responsiveness to environmental factors, together with assessing the potential of serum ALCAM as a marker of prostate cancer progression.

## RESULTS

### Clinical significance of ALCAM in prostate cancer tissues and serum

**Figure 1:**
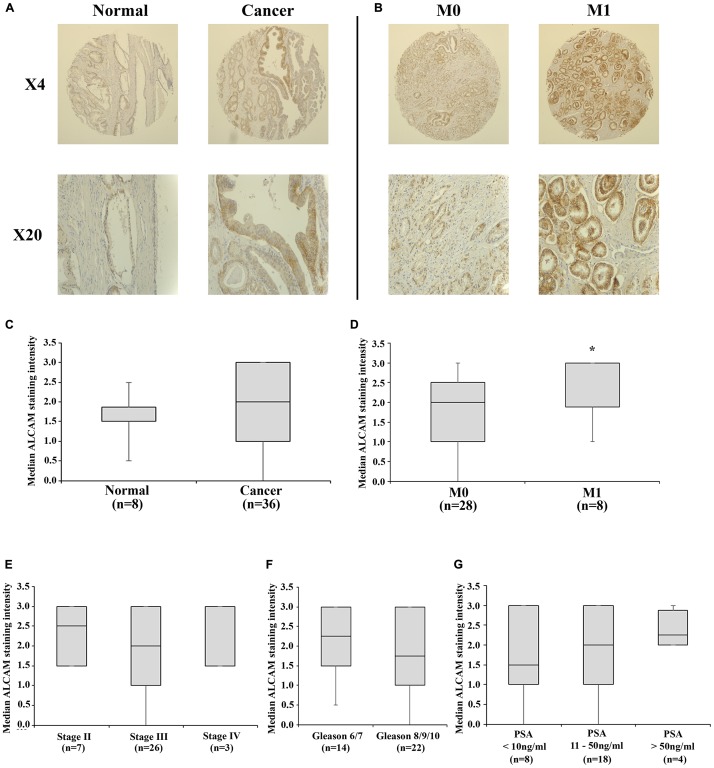
ALCAM tissue expression in a tissue microarray of prostate and prostate cancer tissues. Representative images of normal and cancerous cores (**A**) and cores derived from M0 and M1 patients (**B**) shown at ×4 and ×20 objective magnifications. Median staining intensity scores are presented for normal and cancerous tissue (**C**), M0 and M1 patients (**D**), patient stage (**E**), Gleason score (**F**) and PSA score (**G**). Boxplot data represents the median, Q1 and Q3 staining intensity scores and whiskers represent minimum and maximum values. ^*^Represents *p* < 0.05.

ALCAM expression was examined in a tissue microarray (TMA) containing core biopsies of localized, metastatic disease and paired normal tissues. ALCAM expression was observed mainly in epithelial tissues at both cytoplasmic and membranous locations, though differential staining profiles of cytoplasmic and membranous ALCAM were not performed in the current analysis. Enhanced ALCAM staining intensity was observed in cancerous compared to normal samples, though this was not statistically significant (*p =* 0.32; Figure 1A and 1C). Significantly enhanced ALCAM staining was observed in M1 compared to M0 patients (*p =* 0.027; Figure 1B and 1D), though no significant differences were seen between stage (*p =* 0.161; Figure 1E), Gleason score (*p =* 0.150; Figure 1F) or patient prostate specific antigen (PSA) levels (*p =* 0.668; Figure 1G). Furthermore, comparison of paired normal and cancer tissues (*n =* 8 pairs, Supplementary Figure 1), highlighted enhanced staining in cancer tissues in 6 (75%) of the pairs.

**Figure 2:**
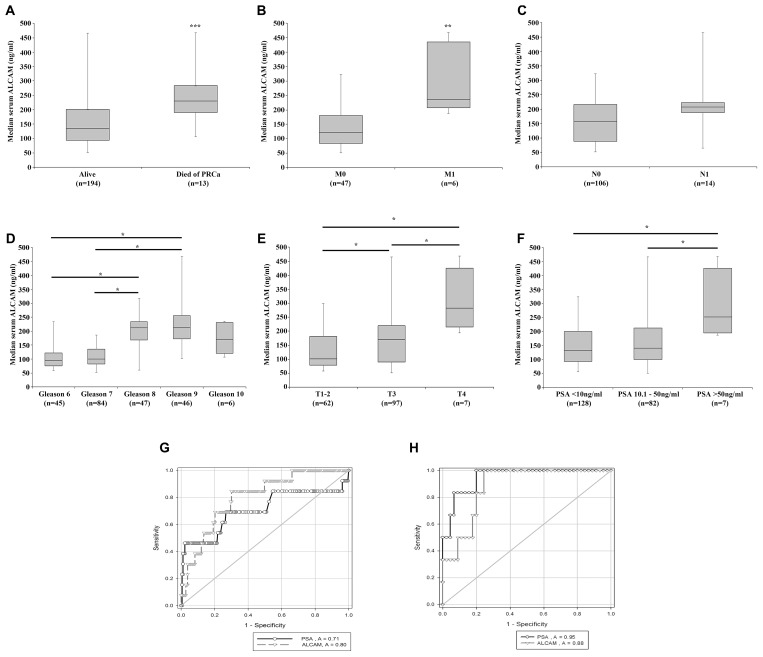
Association of serum ALCAM expression with patient clinical pathological information and prognostic ability. Serum ALCAM levels were examined in a cohort of prostate cancer patients and correlated with available clinical pathological information. Elevated levels of serum ALCAM were observed in patients who died from prostate cancer (**A**), those with detectable metastasis (**B**) and those with nodal involvement (**C**). Higher serum ALCAM was also generally associated with higher Gleason score (**D**), T-score (**E**) and PSA level (**F**). ROC analysis demonstrated a comparable prognostic capacity of serum ALCAM and PSA to predict patients who died of prostate cancer (total *n* = 196 samples with PSA and ALCAM levels; alive, *n* = 183 and died of PRCa, *n* = 13) (**G**) and those with metastasis (total *n* = 51 samples with PSA and ALCAM levels; M0, *n* = 45 and M1, *n* = 6) (**H**). Box plots represent median, Q1 and Q3 data and whiskers represent minimum and maximum values. ^***^Represents *p* ≤ 0.001; ^**^Represents *p* ≤ 0.01; and ^*^Represents *p* < 0.05.

The prognostic potential of serum ALCAM was also assessed in 229 prostate cancer patients (Figure 2). Significantly higher levels of ALCAM were observed in patients who died of prostate cancer (PRCa) compared to those who were still alive (Figure 2A, *p* < 0.001) and in M1 patients compared to M0 patients (Figure 2B, *p =* 0.002), with borderline significant elevations observed in N1 compared to N0 patients (Figure 2C, *p* = 0.05). Significant differences in ALCAM serum levels were observed between Gleason score groups (Figure 2D). Post hoc analysis revealed significantly elevated ALCAM serum levels in Gleason 9 compared to Gleason 7 or Gleason 6 samples, and in Gleason 8 compared to Gleason 7 or Gleason 6 samples (all *p* < 0.05). No significant differences were seen between Gleason 10 and other groups, potentially due to the smaller sample size of this group. Significant differences were also detected between T score (Figure 2E) and PSA level (Figure 2F). Post hoc analysis indicated significantly higher serum ALCAM levels in T4 patients compared to either T3 or a combined T1-2 classification group and in T3 compared to T1-2 samples (all *p* < 0.05) (Figure 2E). Additionally, post hoc analysis indicated significantly elevated serum ALCAM in patients with a PSA level > 50 ng/ml in comparison to those whose PSA < 10 ng/ml and whose PSA was between 10.1 and 50 ng/ml (both *p* < 0.05) (Figure 2F). A positive correlation was also noted between serum PSA and ALCAM levels, where clinical PSA information was available, (*n =* 217, Spearman Rank correlation coefficient = 0.275, *p* ≤ 0.001, data not shown).

Receiver operating characteristic (ROC) analysis was undertaken to explore the prognostic capacity of serum ALCAM, compared to PSA, to identify patients who died of PRCa (Figure 2G) or with metastasis (Figure 2H). Combined ALCAM and PSA serum levels were available for 196 patients with survival outcomes (alive, *n* = 183; died of PRCa, *n* = 13) and 51 patient with M scores (M0, *n* = 45; M1, *n* = 6). Serum ALCAM proved to have comparable diagnostic power to PSA, performing slightly better at identifying those patients who died of PRCa (ALCAM AUC = 0.80, 95% CI 0.69 – 0.91 compared to PSA AUC = 0.71, 95% CI 0.51 – 0.91) (Figure 2G) and slightly worse at identifying those patients with metastasis (ALCAM AUC = 0.88, 95% CI 0.78 – 0.99 vs. PSA AUC = 0.95, 95% CI 0.88 – 1.02) (Figure 2H).

### Generation and verification of ALCAM suppression in PC-3 and LNCaP prostate cancer lines

**Figure 3:**
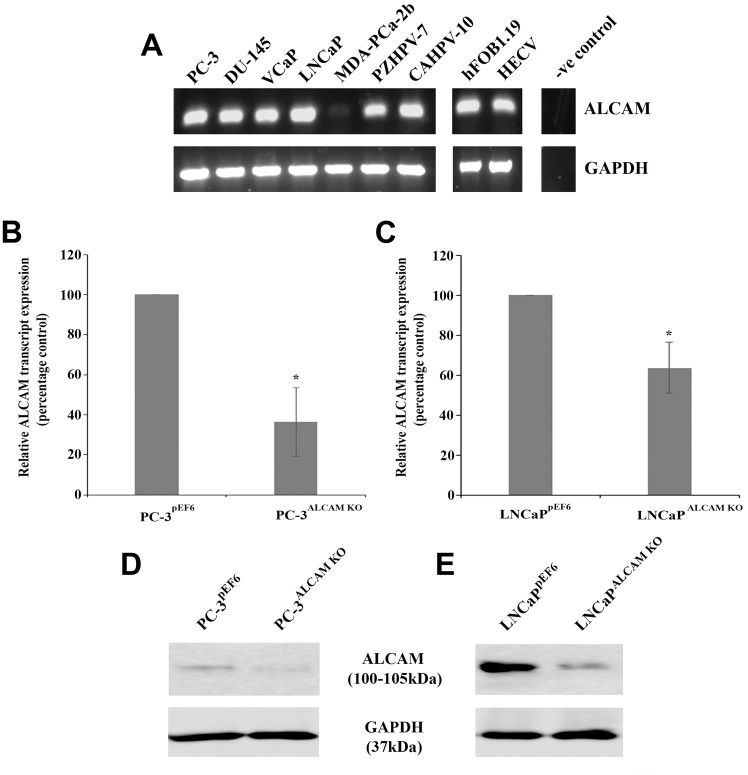
ALCAM expression and targeting in prostate cancer cell lines. RT-PCR demonstrating ALCAM transcript expression in the majority of human prostate cell lines, hFOB1.19 osteoblast and HECV endothelial cell lines (**A**). Quantitative PCR demonstrated that targeting of ALCAM significantly reduced transcript expression in the PC-3 cell line (*n =* 3) (**B**) and the LNCaP cell line (*n =* 3) (**C**). Western blot analysis demonstrating reduced protein levels following ALCAM targeting in the PC-3 (**D**) and LNCaP (**E**) cell lines. Composite gel/blot images show representative images, cropped for conciseness. Comparative bands and adjustments for each individual molecule were taken from the same image. Data represents mean percentage control values ± SEM. ^*^Represents *p* < 0.05.

ALCAM transcript expression was screened in a number of human prostate, prostate cancer, osteoblast (hFOB1.19) and endothelial (HECV) cell lines (Figure 3A). With the exception of the MDA-PCa-2b cell line, ALCAM was expressed at moderate to strong levels. PC-3 and LNCaP cell lines were chosen for transfection with either pEF6 control plasmids or pEF6 plasmids containing ribozyme transgenes targeting ALCAM. Quantitative transcript analysis revealed a significant decrease in ALCAM expression, compared to control cells, in PC-3 cells (*p =* 0.021) and LNCaP cells (*p =* 0.049) following transfection with the ALCAM ribozyme transgene (Figure 3B and 3C). Similarly, western blot analysis indicated a suppression of ALCAM protein in both PC-3 and LNCaP cells transfected with the ALCAM ribozyme transgene (Figure 3D and 3E).

### Functional characterization of ALCAM suppression and impact on cellular responsiveness to a bone-like environment

**Figure 4:**
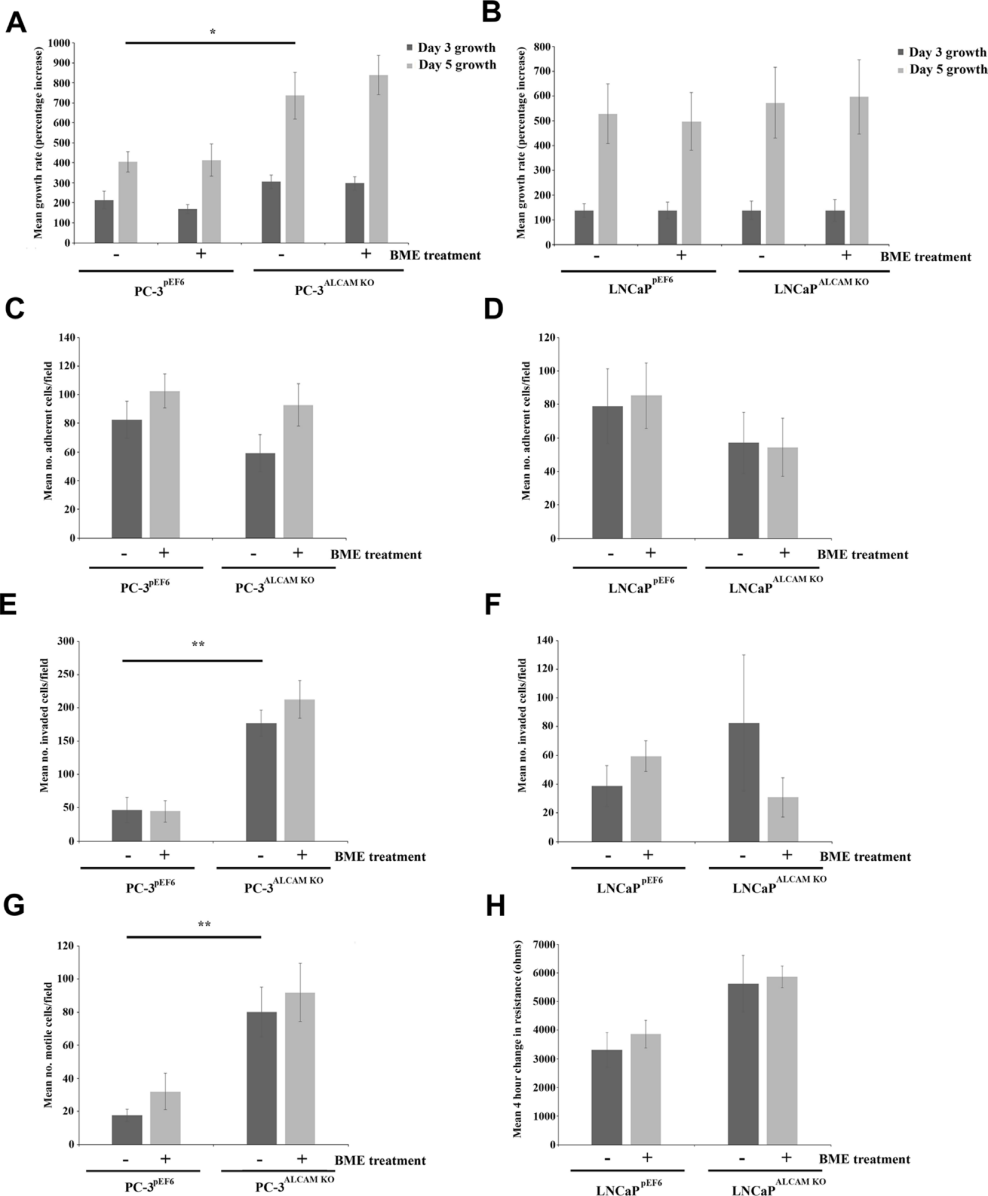
Impact of ALCAM suppression and bone matrix extract (BME) on prostate cancer cell function. Cell growth in response to ALCAM knockdown and treatment with BME in PC-3 (*n =* 4) (**A**) and LNCaP (*n =* 4) (**B**) cell lines. Impact of ALCAM suppression and BME treatment on cell matrix adhesion in the PC-3 (*n =* 3) (**C**) and LNCaP (*n =* 4) (**D**) cell line. Impact of ALCAM suppression and BME treatment on cellular invasion in PC-3 (*n =* 3) (**E**) and LNCaP (*n =* 3) (**F**) cell lines. Cell motility bead assay demonstrating the response of PC-3 cells to ALCAM knockdown and BME treatment (*n* = 4) (**G**). Cell migration in the LNCaP cell line in response to ALCAM suppression and BME treatment following a 4 hour period, quantified using an ECIS based assay (*n* = 4) (**H**). Data represents mean values ± SEM., ^***^Represents *p* ≤ 0.001, ^**^ Represents *p* ≤ 0.01 and ^*^Represents *p* < 0.05.

Functional characterization of ALCAM suppressed cells was undertaken in conjunction with 50 μg/ml bone matrix extract (BME) intended to mimic a bone like environment *in vitro* (Figure 4). Knockdown of ALCAM in untreated PC-3 cells significantly enhanced PC-3 cell growth rates over a 5 day incubation period (*p =* 0.04) but had no significant effect on the LNCaP cell line. BME treatment had no significant impact on the growth of either cell line (Figure 4A and 4B). ALCAM knockdown in untreated cells did not significantly impact on the cell-matrix adhesion of either PC-3 (Figure 4C) or LNCaP cells (Figure 4D), though general reductions were observed in both cell lines. No significant effects were noted following treatment of either line with BME, though generally a greater response was seen in PC-3^ALCAM KO^ cells compared to PC-3^pEF6^ cells. ALCAM knockdown also significantly enhanced cellular invasiveness in untreated PC-3 cells (*p* = 0.009), and non-significantly enhanced LNCaP invasiveness (*p =* 0.427). Differential responses to BME treatment within PC-3 and LNCaP cells was again observed, although significance was not reached (Figure 4E and 4F). Motility bead analysis demonstrated enhanced motility in PC-3^ALCAM KO^ cells compared to PC-3^pEF6^ control cells (*p* = 0.007, Figure 4G) and similarly, LNCaP^ALCAM KO^ cells were found to have enhanced, though non-significantly, 4 hour migratory rates compared to LNCaP^pEF6^ cells using an electric cell substrate impedance sensing (ECIS) based assay (*p* = 0.091; Figure 4H). In both cell lines, the addition of BME had no significant impact on motility.

### Potential mechanistic impact of ALCAM suppression in prostate cancer cell lines

**Figure 5:**
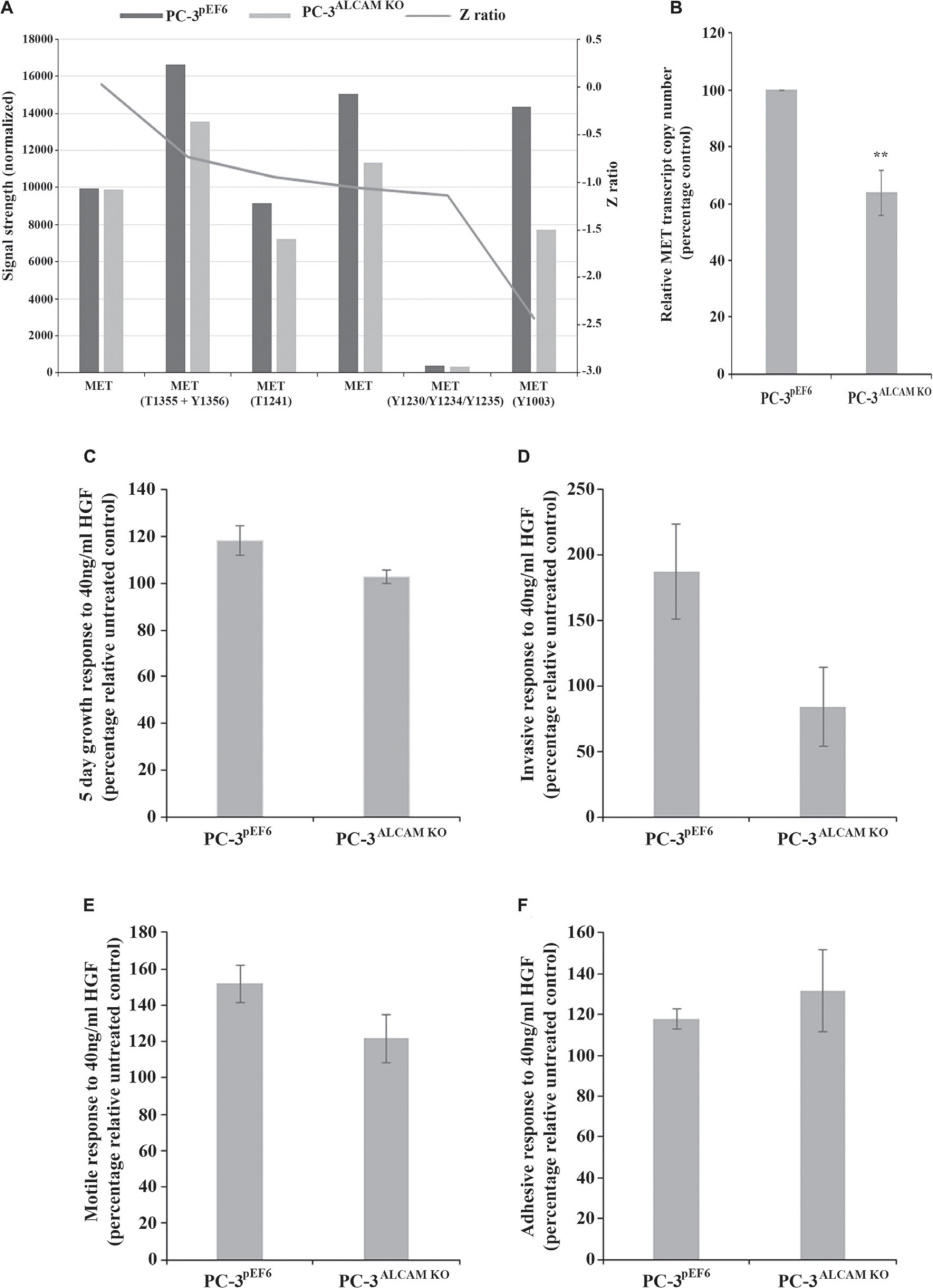
Mechanistic implications of ALCAM suppression and impact on responsiveness to hepatocyte growth factor (HGF). Protein microarray demonstrating signal expression profile of included MET and phospho-MET antibodies in PC-3 control and ALCAM knockdown cells (*n =* 1) (**A**). Quantitative PCR analysis of MET transcript expression in PC-3 control and ALCAM knockdown cells (*n =* 3) (**B**), quantitative PCR data shown represents mean percentage control ± SEM, ^**^ represents p ≤ 0.01. Impact of HGF treatment on the responsiveness of PC-3 control or ALCAM knockdown cells on 5 day growth (*n =* 4) (**C**), invasion (*n =* 3) (**D**), motility (*n =* 4) (**E**) and matrix-adhesion (*n =* 3) (**F**). Data shown represents the mean percentage change ± SEM in comparison to the relative untreated control or ALCAM knockdown cells to demonstrate respective responsive rate to HGF in each individual line.

A protein microarray was used to identify potential differences between PC-3^pEF6^ and PC-3^ALCAM KO^ cells. This highlighted differential expression and/or phosphorylation of certain receptors, including the hepatocyte growth factor (HGF) receptor MET, where a general decrease in signal intensity was observed in a number of pan and phospho-specific probes (Figure 5A). Subsequently, quantification of MET transcript levels indicated a significant reduction in PC-3^ALCAM KO^ compared to PC-3^pEF6^ cells (*p =* 0.01, Figure 5B). We also explored the responsiveness of PC-3^pEF6^ and PC-3^ALCAM KO^ cells to HGF. Data is presented as percentage change in either PC-3^pEF6^ or PC-3^ALCAM KO^ following treatment with 40ng/ml HGF in relation to the relative untreated control to give an indication and comparison of the capacity of such cells to respond to HGF, with >100% indicating a positive and < 100% indicating an inhibitory response. PC-3^ALCAM KO^ cells were generally found to have a lower capacity to respond to HGF than PC-3^pEF6^ cells in terms of 5 day growth (*p =* 0.067, Figure 5C), invasion (*p =* 0.094, Figure 5D) and motility (*p =* 0.126, Figure 5E). No significant differences were seen regarding cell-matrix adhesion (*p =* 0.537, Figure 5F). We further analyzed the expression profile of a number of other key molecules which may act downstream. Within the protein array, alterations were seen in a number of pan and phospho-specific AKT1 antibodies (Supplementary Figure 2A), though both enhanced and decreased total expression was observed across different antibodies following ALCAM knockdown and subsequently, no significant difference in transcript expression was observed (*p* = 0.229; Supplementary Figure 2B) between PC-3^pEF6^ and PC-3^ALCAM KO^ cells. Similarly expression profiles of total and phospho-specific expression of extracellular signal-regulated kinase (ERK) 1 & 2 were explored in the protein microarray where general increases in total ERK1 expression and only minor changes in total ERK2 were observed (Supplementary Figure 2C) following ALCAM suppression, whereas no significant differences in the transcript expression of ERK1 (*p* = 0.520; Supplementary Figure 2D) but a significant decrease in ERK2 transcripts were observed (*p* < 0.001; Supplementary Figure 2E) in PC-3^ALCAM KO^ compared to PC-3^pEF6^ cells. Interestingly, according to the micro-array, the greatest difference following ALCAM suppression, relative to the control, was an enhanced phosphorylation of ERK1/2 Y204. Finally, the protein micro-array also demonstrated alterations in focal adhesion kinase (FAK) expression and phosphorylation following ALCAM suppression, with greatest reductions, relative to the control, seen in total FAK, FAK S732 and FAK S722 (Supplementary Figure 2F), which is partially supported at the transcript level where ALCAM suppression led to a significant decrease in FAK expression (*p =* 0.002; Supplementary Figure 2G).

### Impact of ALCAM-Fc chimera on endothelial attachment and function

**Figure 6:**
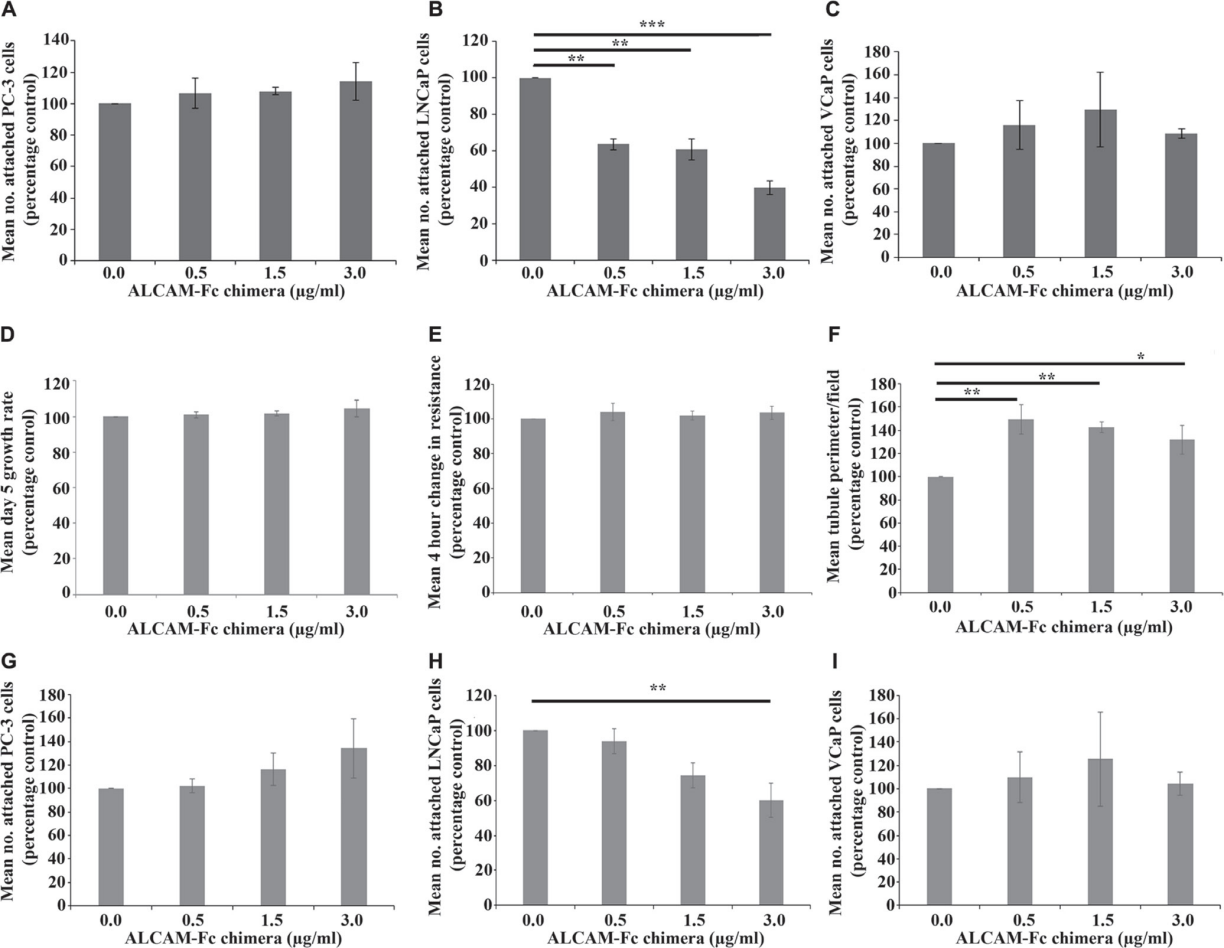
Impact of ALCAM-Fc chimera on cancer cell attachment and endothelial cell function. Effect of increasing concentrations of ALCAM-Fc chimera on PC-3 (**A**), LNCaP (**B**) and VCaP (**C**) cancer cell attachment to a HECV endothelial monolayer. Impact of increasing concentrations of ALCAM-Fc chimera on 5 day growth rates (**D**), 4 hour migration (**E**) and tubule formation (**F**) of HECV human endothelial cells. Change in resistance during an ECIS based wounding assay was taken as a surrogate of cellular migration. Effect of increasing concentrations of ALCAM-Fc chimera on PC-3 (**G**), LNCaP (**H**) and VCaP (**I**) cancer cell attachment to an hFOB1.19 osteoblast monolayer. Data shown represents mean percentage control (*n =* 3) ± SEM. ^***^Represents *p* ≤ 0.001, ^**^Represents *p* ≤ 0.01 and ^*^Represents *p* < 0.05.

The impact of an ALCAM-Fc chimera, containing Trp28-Ala526 of ALCAM, on prostate cancer cell attachment to HECV endothelial cells was explored. No tested concentration of ALCAM-Fc chimera significantly impacted on PC-3 (Figure 6A) or VCaP (Figure 6C) attachment to the HECV monolayer. However, significant differences were seen on LNCaP attachment (Figure 6B) with post hoc analysis indicating significant inhibition at 0.5 μg/ml (*p* = 0.002), 1.5 μg/ml (*p* = 0.002) and 3.0 μg/ml (*p* < 0.001) concentrations compared to untreated control.

ALCAM-Fc chimera did not significantly influence HECV 5 day growth rates (Figure 6D) or cellular migration (Figure 6E) but significantly impacted on HECV tubule formation, with post hoc analysis highlighting significant increases at 0.5 μg/ml, 1.5 μg/ml and 3.0 μg/ml concentrations (*p* = 0.005, 0.01 and 0.038, respectively, vs. untreated HECV cells) (Figure 6F).

### Impact of ALCAM-Fc chimera on osteoblast attachment

No significant alterations were seen in PC-3 or VCaP attachment to a hFOB1.19 osteoblast monolayer at any ALCAM-Fc chimera concentration (Figure 6G and 6I), though generally higher numbers of PC-3 cells adhered at the 3.0 μg/ml concentration. Increasing concentrations of ALCAM-Fc chimera were found to significantly inhibit LNCaP attachment rates (Figure 6H), with post hoc analysis highlighting significant attachment inhibition at 3.0 μg/ml concentration (*p =* 0.007 vs. untreated control).

## DISCUSSION

The cell adhesion molecule ALCAM has been recognized as an important molecule in cancer progression and metastasis though its precise role appears to be complex and remains to be fully elucidated. The current study aimed to further explore the relevance of ALCAM in prostate cancer cells and associated mechanisms, the potential of serum ALCAM to act as a biomarker of prostate cancer and the impact of an ALCAM-Fc chimera, containing extracellular regions of ALCAM, to influence other cell types involved in the metastatic cascade.

Several studies have looked at ALCAM immunostaining in prostate cancer. Kristiansen *et al.,* highlighted a general up-regulation of ALCAM in tumor tissues, where enhanced expression was associated with low Gleason grade and a lower expression associated with higher grade disease [20]. A later study by this group supported ALCAM over-expression in prostate cancer samples and also demonstrated the potential of cytoplasmic, but not membranous, ALCAM to act as a prognostic marker of PSA relapse, highlighting the significance of protein location in addition to expression in cancer progression [21]. Further work, using a large scale tissue microarray format, noted ALCAM expression predominantly at the membrane with any cytoplasmic staining correlating with strong membranous expression and similarly associated high membranous ALCAM immunostaining to correlate with favorable tumor characteristics such as T stage, nodal status and preoperative PSA, with high ALCAM expression also linked to reduced risk of biochemical recurrence [22]. Differential expression of ALCAM has been observed in many other cancer types, where its cellular location again has been implicated as a significant factor. For example, in breast cancer reduced ALCAM transcript expression has been observed to associate with more aggressive phenotypes and poorer patient outlooks with reductions of either cytoplasmic and/or membranous ALCAM staining observed in patient tumor tissue and/or those with bone metastasis [13, 14]. Another study associated enhanced cytoplasmic ALCAM expression with reduced disease free survival rates, suggesting a role for strong cytoplasmic ALCAM expression as a marker of aggressive disease [15], whereas evaluation of membranous staining in a different study reported a link between decreased ALCAM expression and advanced tumor size, grade, negative estrogen and progesterone status and poorer survival rates, noting links between cytoplasmic staining and stronger membranous staining [12]. A further study, not differentiating between membranous and cytoplasmic staining, demonstrated the correlation of strong ALCAM expression with nodal involvement and presence of tumor cells in the bone marrow, with strong ALCAM expression in ductal carcinomas also correlating with reduced rates of recurrence free and overall survival [7]. In colorectal cancer, analysis of cytoplasmic and membranous ALCAM expression indicated a significant correlation between membranous ALCAM expression and reduced patient survival rates [23]. A different study noted predominant membranous ALCAM expression in colorectal samples, with cytoplasmic staining associated with strong membrane expression, and following the analysis of membranous ALCAM expression reported a significantly reduced overall survival rate in ALCAM negative patients [24]. Similar links to cellular localization have also been implicated in lung cancer, where membranous ALCAM expression, but not cytoplasmic, associated with shortened overall survival [25], oral squamous cell carcinomas (OSCCs) where cytoplasmic accumulation of ALCAM was indicative of poor patient prognosis [26] and pancreatic cancer, where cytoplasmic ALCAM expression in cancer cells was observed compared to membranous expression in normal cells and increased ALCAM expression was linked to adverse recurrence free and overall survival rates [27]. Taken together, ALCAM expression appears to be useful as a predictive prognosis tool, however, some contrast appears within the literature and its role may be dependent on cellular localization or cancer type. In our current study ALCAM staining levels were found to be generally enhanced in cancer tissues and were significantly associated with metastatic disease, though no significant associations were seen with other clinical pathological factors. However, the data presented here represents only a small microarray and no differential expression analysis was performed for membranous or cytoplasmic expression. Hence, these factors may account for discrepancies observed between the results presented here and other studies but again give an indication of the potential use for ALCAM staining as a prognostic factor.

Currently, there is very little information regarding the clinical relevance of serum ALCAM as a prognostic factor for prostate cancer progression. Previous mouse models utilizing human PC-3 and LNCaP xenografts have demonstrated a role for tumor, but not host derived ectodomain ALCAM in influencing the tumor burden of subcutaneous and orphotopic xenographs [6]. Within our cohort, significantly higher levels of serum ALCAM were associated with T stage, Gleason score and survival. ALCAM and PSA levels were also found to correlate and demonstrated comparable abilities to identify patients with metastasis and those who died of the disease, though serum ALCAM is not prostate specific and hence any clinical benefit may be in conjunction with other markers. Numerous studies have explored serum ALCAM in other cancers types where elevated levels are indicative of cancer or of poorer prognosis in gastric cancer [18, 28], hepatocellular carcinoma [29] and esophageal cancer [30]. Similar observations have also been made in breast cancer, where studies have shown ALCAM to perform as well or better than cancer antigen 15-3 (CA15-3) and carcinoembryonic antigen (CEA) [31–33] and in epithelial ovarian cancer, where correlations between serum ALCAM and cancer antigen 125 (CA125) were noted [34]. However, other studies have also suggested either no difference between pancreatic cancer and chronic pancreatitis [35], or that enhanced serum ALCAM may be associated with moderate, compared to poorly differentiated cervical cancer [36]. Currently, PSA testing is widely used in diagnosis and monitoring of prostate cancer, though such a test carries both advantages and limitations and hence there is a need to identify, refine or develop new diagnostic and predictive assays for prostate cancer. Our study demonstrates the significance of serum ALCAM as a potential marker of prostate cancer progression, highlighting its use as a possible indicator of poor prognosis. Further work on larger cohorts of prostate cancer patients are needed to further realize the potential of serum ALCAM, potentially in conjunction with other markers, as a biomarker for prostate cancer and disease progression.

To further explore the wider implications and significance of extracellular ALCAM we explored the impact of an ALCAM-Fc chimera, including Trp28 – Ala526 and representing extracellular regions of ALCAM. The presence of this ALCAM-Fc chimera was found to enhance the tubule formation capacity of HECV human endothelial cells, potentially indicating a pro-angiogenic effect, which could support advanced tumor growth and aid metastatic dissemination. ALCAM has previously been associated with the processes of embryonic hematopoiesis and vasculoangiogenesis and, somewhat in contrast to our data, a soluble ALCAM-Fc chimera has previously been shown to negatively influence tube formation in yolk sac derived endothelial cells [37]. Soluble recombinant human ALCAM-Fc has also been shown to inhibit the trans-endothelial migration of monocytes without influencing migration or attachment to PMVEC endothelial cells [38]. In our current study ALCAM-Fc chimera also had differential effects on cancer cell attachment to endothelial or osteoblast cells, inhibiting LNCaP but not PC-3 or VCaP cell attachment. This may in part be due to differing cellular expression profiles of ALCAM within these cell lines, which could possibly allow for a greater disruption of ALCAM-ALCAM interactions in these cells. However, additional mechanisms, receptors or binding partners may exist and the nature and heterogeneity of a particular cancer type may also be a key factor in such mechanisms.

Our current data suggests that suppression of ALCAM may be associated with a more aggressive cellular phenotype *in vitro*, but this appeared to be largely cell line specific. Many differences exist between PC-3 and LNCaP cells utilized in this study, with perhaps one of the more significant being their androgen receptor (AR) status and responsiveness to androgen. Such differences as well as others may likely account for differential results between the cell lines though the importance of AR or androgen was not directly explored in this study and requires additional scientific investigation. Similar observations regarding ALCAMs role in regulating cellular phenotype have been noted in a number of other studies/cancers, though others suggest a contrasting role for ALCAM in regulating such traits *in vitro* and *in vivo* [6–11]. A recent study by Devis *et al.* [8], demonstrated ALCAM suppression in endometrial cells could reduce migration and invasion *in vitro* and also reduce primary tumor development and metastatic local spread in an orthotopic model, a trait not associated with proliferative changes but suggested to arise through the influence of ALCAM in cell micro-environment communications. Furthermore Devis *et al.,* describe differences in gene profiles between control and ALCAM suppressed cells highlighting a number of pathways altered in cancer, including integrin signaling, as well as genes associated with motility and invasion [8]. ALCAM has also previously been shown to be a key regulator of prostate cancer dissemination to, and tumor development within the bone, with ALCAM suppression reducing skeletal metastasis and intratibial tumor growth but having no proliferative effects on tumor development within an orthotopic model implanted into the prostate [6]. *In vitro* ALCAM suppression has also been shown to result in a loss of TGFβ induced migration but only result in a non-significant increase in spontaneous migration rates [6]. To further explore potential mechanisms involved we undertook a protein microarray. Interestingly, differential total- or phospho-expression was seen in a number of receptors, signaling pathways and downstream effectors. This may provide a clue as to the differential *in vitro* and clinical impact of ALCAM in our study, and may suggest that loss of ALCAM may influence cellular traits but may also regulate the expression/phosphorylation of receptors and/or signaling pathways such as MET, a pathway frequently dysregulated in cancer [39], and/or other downstream effector pathways. However, the data presented is preliminary and some contrast exists between protein data within the micro-array and/or the quantitative transcript analysis. Similarly, loss or reduced expression of molecules such as FAK have previously been associated with a less aggressive cancer type and inhibition/disruption of this pathway as a potential therapeutic [40], rather than a more aggressive cellular phenotype reported here. Taken together, this suggests ALCAM may act as a mediator of tumor cell interactions with the surrounding micro-environment and a regulator of signal propagation within the cell, though this appears to be dependent on the particular environment or factors present. However, additional intense study is required to fully explore this, focusing on validation of the micro-array using conventional methods, together with exploring the impact of ALCAM suppression in complex multi-cellular environments. Furthermore, our data provides additional relevance to the potential roles played by extracellular ALCAM which may impact angiogenesis or cell attachment at secondary sites. Together, this may help explain the differential, complex role of ALCAM at a cellular and wider clinical level and supports the dual role of ALCAM proposed by Hansen *et al.* [6]. Further intense work is necessary to fully elucidate these complex roles and the clinical potential of ALCAM.

In summary, our current data suggest that serum ALCAM may have promise as a prognostic indicator in prostate cancer. Furthermore, ALCAM may influence cellular traits, and also their responsiveness to external stimuli. Extracellular ALCAM may also contribute to aspects of metastatic dissemination.

## MATERIALS AND METHODS

### Cell lines, materials and culture conditions

PC-3, LNCaP, VCaP, CAHPV-10, PZHPV-7, hFOB1.19, MDA-PCa-2b and DU-145 cell lines were purchased from the American Type Culture Collection (ATCC; Middlesex, UK). Human HECV endothelial cells were purchased from Interlab Cell Line Collection (ICLC; Genoa, Italy). PC-3, LNCaP, VCaP, HECV and hFOB1.19 cells were used in functional assays. PC-3, VCaP and HECV cells were grown in Dubecco’s Modified Eagle Medium (DMEM)/Ham’s F12 with L-glutamine (Sigma-Aldrich, Dorset, UK) and LNCaP cells were cultured in RPMI 1640 medium (Sigma-Aldrich, Dorset, UK). Human hFOB1.19 osteoblasts were maintained in DMEM/Ham’s F-12 without phenol red (Life Technologies, Paisley, UK) containing 0.3mg/ml G418 (Melford Laboratories, Suffolk, UK). All base mediums were supplemented with 10% foetal calf serum (FCS) (Sigma-Aldrich, Dorset, UK) and an antimicrobial solution (Sigma-Aldrich, Dorset, UK). Cells were cultured at 95% humidity, 5% CO_2_ and 37° C except hFOB1.19 which were cultured at 34° C.

HGF was a kind gift from Dr T Nakamura (Osaka University Medical School, Japan). Recombinant human ALCAM, comprising a fusion of ALCAM Trp28 - Ala526 and the human IgG Fc region (ALCAM-Fc chimera) was purchased from R&D systems (Abingdon, UK).

### Preparation of bone matrix extract (BME) from femoral heads

BME was generated in house from human femoral heads and has been previously described [11]. Briefly, femoral heads, obtained immediately following hip replacement surgery in conjunction with ethical approval, were ground using a bone mill (Splerings Orthopaedics B. V., The Netherlands). Fine material was collected in balanced saline solution (BSS) and subjected to sonication at 4° C using a BioRuptor (Wolf Laboratories, York, UK) and the resulting solutions collected and stored at –80° C.

### Clinical samples

A TMA comprising 48 patient samples, containing duplicate cores from 36 patients diagnosed with adenocarcinoma and duplicate (with the exception of 1 sample, where *n =* 1) cores of matched normal tissue for 8 patients, (HPro-Ade96Sur-01) was purchased from Insight Biotechnologies (Middlesex, UK). Single cores from 4 metastatic locations were also contained on the array though due to the nature of these tissue types, namely bone section staining, were not included in the analysis. Where available, the median follow up period was 23 months.

Serum samples from prostate cancer patients were obtained from the Wales Cancer Bank (WCB) which is funded by the Wales Assembly Government and Cancer Research Wales. Other investigators may have received specimens from the same subjects. A total of 229 samples were utilized in the study with a median patient age of 65 years and a median follow up period of 4.0 years.

### Immunohistochemical staining of ALCAM in a prostate cancer TMA

ALCAM expression was assessed across a TMA of normal prostate and prostate cancer sections. TMA antigen retrieval was undertaken in 0.1M EDTA buffer, heated in a microwave for 20 minutes, allowed to cool under running tap water and blocked with 5–10% horse serum for two hours. Following blocking, the TMA was incubated overnight with ALCAM primary antibody (final concentration 2 μg/ml; Novacastra, Milton Keynes, UK) before incubation with secondary and tertiary reagents from a Vectastain Elite Universal ABC kit (Vector Laboratories ltd., Peterborough, UK), in accordance with the manufacturers guidelines, and developing with diaminobenzidine (5mg/ml; Sigma-Aldrich, Dorset, UK) for 10 minutes. Subsequently, the TMA was counterstained with Gill’s hematoxylin (Vector Laboratories ltd., Peterborough, UK), dehydrated, cleared in xylene and mounted in DPX (Sigma-Aldrich, Dorset, UK). Digital images were acquired under the microscope and epithelial staining intensity assessed and scored by three researchers as no (0), weak (1), moderate (2), or strong (3). Where initial conflict occurred, samples were reanalyzed and a consensus decision reached. Average scores for duplicate cores were used.

### Enzyme linked immunosorbent assay (ELISA) quantification of serum ALCAM

Patient serum ALCAM was detected using a human ALCAM (CD166) ELISA assay (Life Technologies, Paisley, UK) in accordance with the manufacturer’s guidelines and analyzed in conjunction with available clinicopathological information.

### Generation of ALCAM suppression in prostate cancer cells

ALCAM specific ribozyme transgenes were designed, generated and cloned as described in previous studies [11, 41]. Plasmids were transfected into mammalian cells using electroporation. Both plasmids containing ribozyme transgenes (designated ^ALCAM KO^) and control plasmids (designated ^pEF6^) were used to transfect cells.

### RNA extraction and reverse transcription polymerase chain reaction (RT-PCR)

RNA was extracted using TRI-reagent (Sigma-Aldrich, Dorset, UK) in accordance with the manufacturer’s instructions. Following extraction, RNA was standardized and reverse transcribed using a high capacity cDNA reverse transcription kit (Life Technologies, Paisley, UK). Subsequently, polymerase chain reaction (PCR) was undertaken using primers designed to amplify Glyceraldehyde 3-phosphate dehydrogenase (GAPDH) or ALCAM (Table 1), GoTaq Green Mastermix (Promega UK, Southampton, UK), sample cDNA and molecular biology grade water. PCR conditions were; initial denaturing at 94° C for 5 minutes followed by 32–34 cycles of 94° C for 30 seconds, 55° C for 30 seconds and 72° C for 40 seconds, before a final extension of 72° C for 10 minutes and holding at 4° C. Products were subsequently separated on an agarose gel stained with SYBR safe (Life Technologies, Paisley, UK) and visualized under blue light.

**Table 1:** Primers used in the study

Primer	Forward	Reverse
ALCAM (PCR)	TTATCATACCTTGCCGACTT	GGGTGGAAGTCATGGTATAG
ALCAM (qPCR)	CAGGAGGTTGAAGGACTAAA	ACTGAACCTGACCGTACAGGGATCAGTTTTCTTTGTCA
GAPDH (PCR)	GGCTGCTTTTAACTCTGGTA	GACTGTGGTCATGAGTCCTT
GAPDH (qPCR)	AAGGTCATCCATGACAACTT	ACTGAACCTGACCGTACAGCCATCCACAGTCTTCTG
MET (qPCR)	ACTGAACCTGACCGTACAGAGCCAAAGTCCTTTCAT	ATCGAATGCAATGGATGAT
FAK (qPCR)	CTATCCAGGTCAGGCATCT	ACTGAACCTGACCGTACACGCAGGTCCAATACTGTAGA
AKT (qPCR)	CTACTACGCCATGAAGATCC	ACTGAACCTGACCGTACAGGTCTGGAAAGAGTACTTCAG
ERK1 (qPCR)	ACACGCAGTTGCAGTACA	ACTGAACCTGACCGTACAGGGGCTGATCTTCTTGAT
ERK2 (qPCR)	CCAACCTCTCGTACATCG	ACTGAACCTGACCGTACAGGGGCTGATTTTCTTGAT

*ACTGAACCTGACCGTACA* represents *Z* sequence.

### Quantitative polymerase chain reaction

Quantitative polymerase chain reaction (qPCR) was undertaken based on a previously reported technique [42, 43]. Briefly, reactions were prepared containing, PrecisionFAST qPCR mastermix (Primer Design, Eastleigh, UK), forward primer, z-tagged reverse primer (Table 1), Uniprimer probe (Intergen Inc., Oxford, UK), molecular biology grade water and sample cDNA and were run on a StepOne Plus qPCR detection system (Life Technologies, Paisley, UK). Reaction conditions were; initial 95° C for 15 minutes followed by 100 cycles of 95° C for 15 seconds, 55° C for 35 seconds and 72° C for 20 seconds. Samples were run simultaneously with a standard of known transcript copy number, allowing calculation of relative transcript expression. Quantification of GAPDH expression was subsequently used to normalize samples.

### Sodium dodecyl sulfate-polyacrylamide gel electrophoresis (SDS-PAGE) and western blotting

Cells were harvested and lysed in lysis buffer for 1 hour, on a rotating wheel at 4° C, before being centrifuged at 13,000g to remove insolubles, quantified using a Bio-Rad DC protein assay kit (Bio-Rad laboratories, Hemel Hempstead, UK), standardized, diluted in sample buffer (Sigma-Aldrich, Dorset, UK) and boiled for 5 minutes. Samples were separated using SDS-PAGE and transferred to an Immobilon-P PVDF membrane (Sigma-Aldrich, Dorset, UK) using a semi-dry method. Membranes were probed using a SNAP-ID system (Merck-Millipore, Watford, UK) in accordance with the manufacturer’s guidelines and visualized using EZ-ECL reagent (GeneFlow, Lichfield, UK). ALCAM and GAPDH primary antibodies (Insight Biotechnology ltd., Middlesex, UK) and an anti-mouse HRP conjugated secondary antibody (Sigma-Aldrich, Dorset, UK) were used to detect proteins of interest.

### Cell characterization assays

A range of cell characterization assays were performed to assess the impact of ALCAM on cellular functions based on previously described methods [42, 44, 45]. Briefly, cell growth rates were assessed using an *in vitro* cell growth assay. Cells were seeded into triplicate 96 well plates and incubated for either overnight, 3 days or 5 days. Following incubation cells were fixed in 4% formalin and stained in 0.5% crystal violet before extracting stain with 10% acetic acid and measuring absorbance at 540nm, allowing the calculation of percentage growth increase from the overnight reference plate.

Cellular invasion was assessed using a Matrigel invasion assay. PC-3 and LNCaP cells were seeded into transwell inserts, containing 8.0μm pores, pre-coated with 50 μg of Matrigel (Corning, UK) suspended in a 24 well plate containing growth media. Following 3 day incubation, inserts were removed and the inner chamber cleaned thoroughly before fixing cells on the underside of the insert in 4% formalin and staining with 0.5% crystal violet. Representative images were then captured and quantified under ×20 objective magnification.

A matrix adhesion assay was used to assess cellular attachment to Matrigel. Cells were seeded into 96 well plates pre-coated with 5 μg/ml Matrigel and incubated for 45 minutes. Subsequently, wells were washed in PBS and adherent cells fixed in 4% formalin and stained with 0.5% crystal violet. Representative images were then captured and quantified under ×20 objective magnification.

PC-3 cell motility was assessed using an *in vitro* cytodex-2 bead motility assay. Cells were seeded into 10ml of growth medium containing cytodex-2 beads and incubated overnight. Subsequently, the beads were washed, pelleted and resuspended before seeding into a 96 well plate. Following 4 hour incubation, plates were washed with PBS, fixed in formalin and stained with crystal violet. Representative images were taken, and quantified, of cells which had migrated to the bottom of the well under ×20 objective magnification.

LNCaP and HECV cell migration was assessed using an ECIS assay using an ECIS Zθ system and 96W1E 96 plates (Applied Biophysics Inc., NY, USA). Cells were seeded and incubated to allow confluence before inducing a wound by applying 1400uA for 30 seconds (for LNCaP cells) or 3000uA for 20 seconds (for HECV cells). The change in resistance within each well was subsequently recorded and used to quantify migration.

Angiogenic potential was assessed using a Matrigel tubule formation assay. Briefly, 96 well plates were coated with 500 μg of Matrigel before seeding HECV cells and incubating for 4-6 hours. Tubules formed over this period were visualized under ×4 objective magnification and quantified through assessment of total tubule perimeter per field.

### Kinexus protein microarray analysis

Differences in total and phospho-protein expression between PC-3^pEF6^ and PC-3^ALCAM KO^ were assessed using a Kinexus protein microarray as previously described [46]. Briefly, harvested cells were resuspended and lysed in lysis buffer for 1 hour, on a rotating wheel at 4° C, insolubles removed, through centrifugation, and samples standardized before being sent to Kinexus Bioinformatics, Vancouver, Canada for analysis using a KAM-880 array. Differential expression between PC-3^pEF6^ and PC-3^ALCAM KO^ samples was interrogated based on normalized signal strength and Z ratio.

### *In vitro* 1,1’-Dioctadecyl-3,3,3′,3′-Tetramethylindocarbocyanine Perchlorate (DiI) cell-cell interaction assays

Cell interaction/attachment was assessed in the presence of ALCAM-Fc chimera using a DiI staining technique based on a previously described method [47]. Briefly, HECV or hFOB1.19 cells were grown to confluence in a 96 well plate before seeding cancer cells, pre-stained with 5μM DiI for 30 minutes, onto the monolayer and incubating for 40 minutes. Subsequently, wells were washed in PBS and adherent cells fixed in 4% formalin. Representative bright field and TRITC images were captured on a Leica fluorescent inverted microscope (Leica Microsystems Ltd., Milton Keynes, UK) at ×20 objective magnification, the images merged and attached cancer cells quantified.

### Statistical analysis

Statistical analysis was undertaken using the SigmaPlot 11.0 (Systat Software Inc., London, UK) and Minitab 14 (Minitab Ltd., Coventry, UK) statistical software packages and data analyzed using a t-test, Mann Whitney test, one way ANOVA or Kruskal-Wallis ANOVA on RANKS with post hoc analysis. *p* < 0.05 was considered to be statistically significant. ROC and Spearman’s rank correlation tests were used to analyze patient serum data.

## SUPPLEMENTARY MATERIALS



## Abbreviations

ADAM: a disintergrin and metalloprotease
ALCAM: activated leukocyte cell adhesion molecule
AR: androgen receptor
BME: bone matrix extract
CA125: cancer antigen 125
CA15-3: cancer antigen 15-3
CEA: carcinoembryonic antigen
ECIS: electric cell substratum impedance sensing
EGF: epidermal growth factor
ELISA: enzyme linked immunosorbent assay
ERK: extracellular signal regulated kinase
FAK: focal adhesion kinase
GAPDH: glyceraldehyde-3-phosphate dehydrogenase
HGF: hepatocyte growth factor
PRCa: prostate cancer
PSA: prostate specific antigen
ROC: receiver operating characteristic
TGFβ: transforming growth factor beta
TMA: tissue microarray
