# Supplementary material for: Importance of activated leukocyte cell adhesion molecule (ALCAM) in prostate cancer progression and metastatic dissemination
Source: Oncotarget. 2019 Oct 29;10(59):6362–77. doi: 10.18632/oncotarget.27279 (PMC6824871; doi:10.18632/oncotarget.27279)
Supplement: Supplementary file 1 [file oncotarget-10-6362-s001.pdf]

Importance of activated leukocyte cell adhesion molecule (ALCAM) in prostate cancer progression and metastatic dissemination

SUPPLEMENTARY MATERIALS

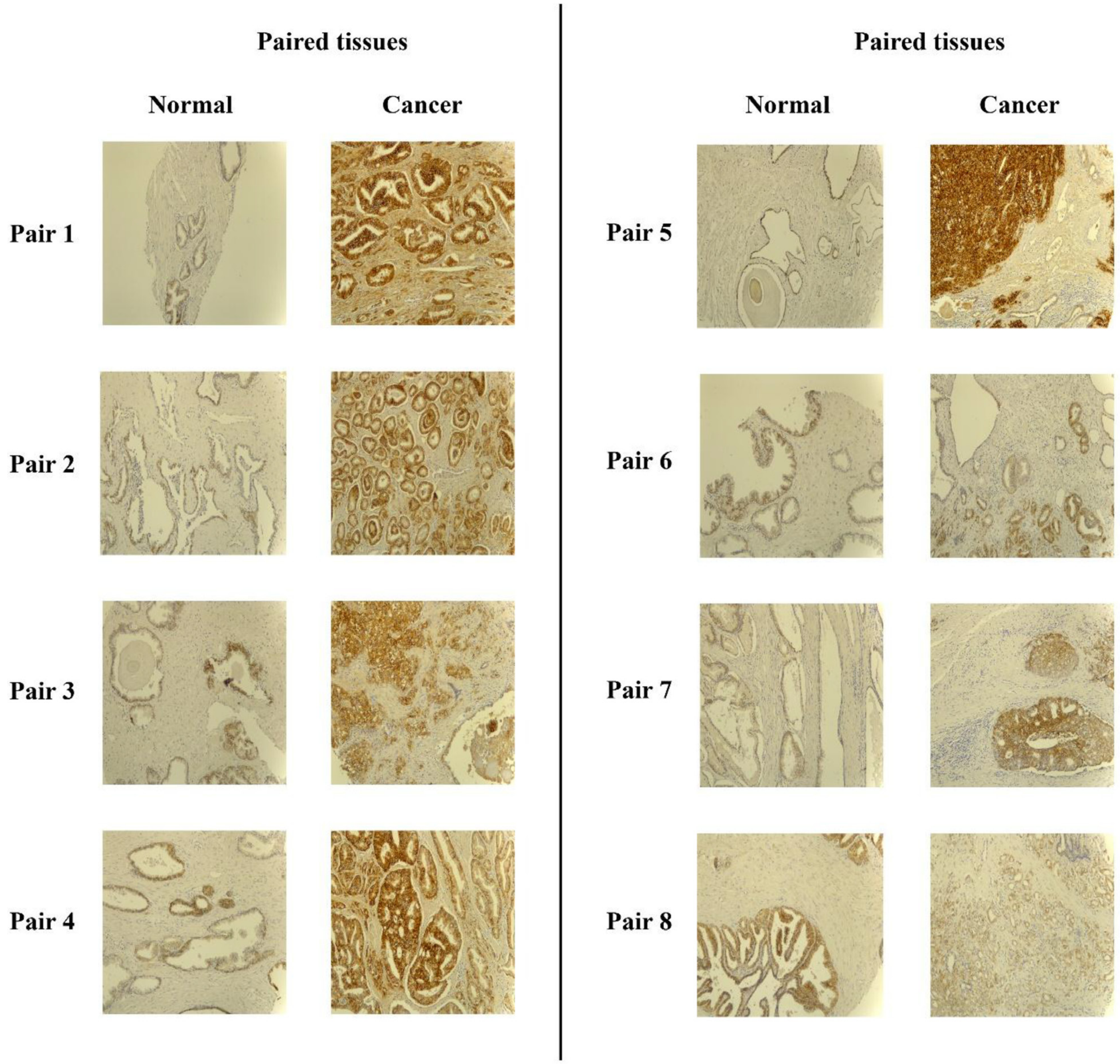

Supplementary Figure 1: ALCAM staining intensity in paired normal and cancerous tissues. Representative images of X10 objective magnification shown. Analysis of paired tissue was undertaken in conjunction to larger patient cohort analysis and hence some sections are presented again for direct comparison with associated pair.

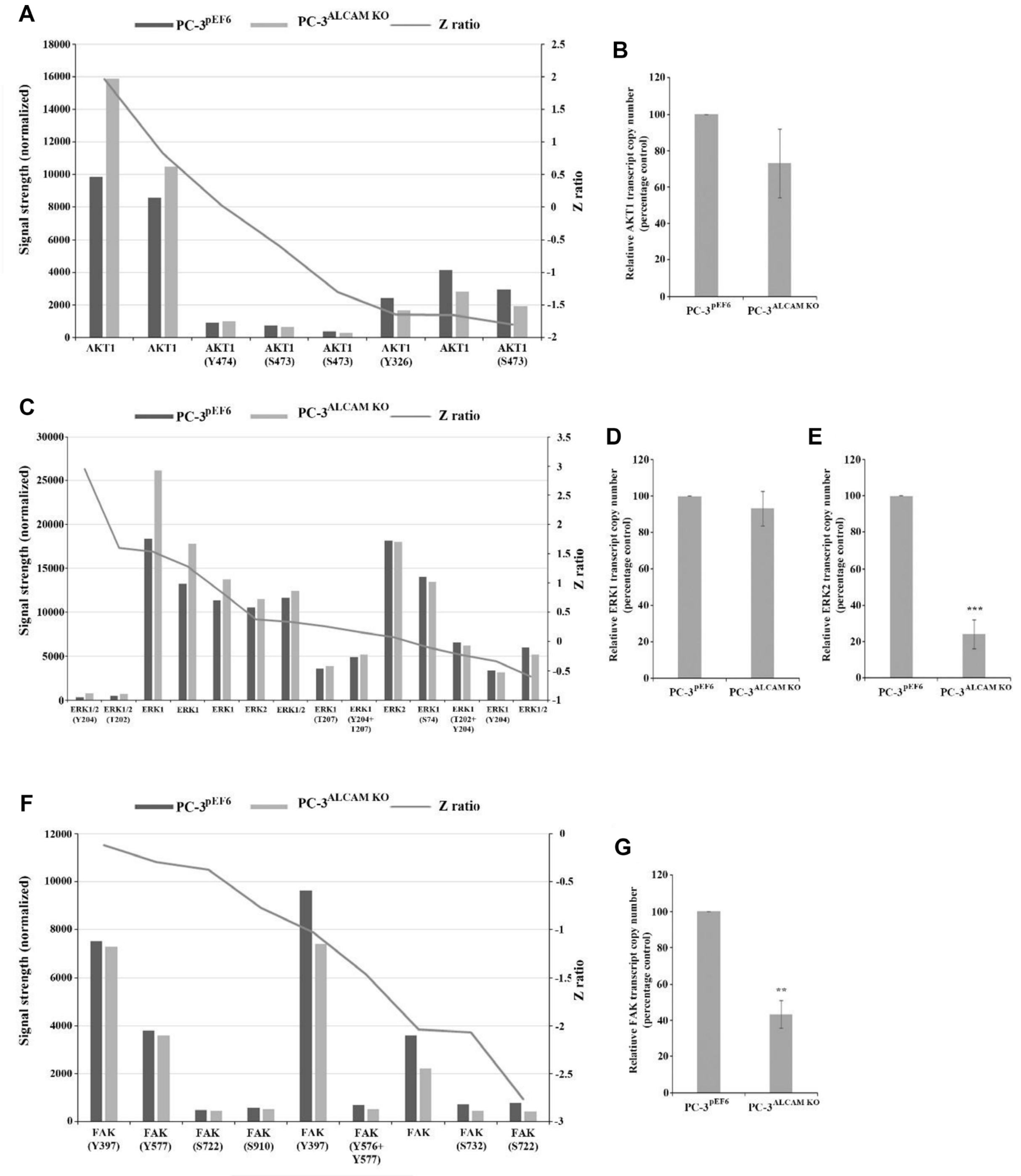

Supplementary Figure 2: Differential expression of potential downstream molecules following ALCAM suppression in PC-3 cells. Protein microarray analysis of pan-specific and phospho-specific AKT1 levels (n = 1) (**A**) and quantitative PCR analysis of AKT1 transcript expression (n = 3) (**B**) in PC-3^{pEF6} and PC-3$^{ALCAM\ KO}$ cells. Protein microarray analysis of pan-specific and phospho-specific ERK1 and 2 levels (n = 1) (**C**) and quantitative PCR analysis of ERK1 (n = 3) (**D**) and ERK2 (n = 3) (**E**) transcript expression in PC-3^{pEF6} and PC-3$^{ALCAM\ KO}$ cells. Protein microarray analysis of pan-specific and phospho-specific FAK levels (n = 1) (**F**) and quantitative PCR analysis of FAK transcript expression (n = 3) (**G**) in PC-3^{pEF6} and PC-3$^{ALCAM\ KO}$ cells. Quantitative PCR data shown represents the mean percentage control value ± SEM ** represents $p \leq 0.01$, *** represents $p \leq 0.001$.
